# Deletion of Lipoprotein *PG0717* in *Porphyromonas gingivalis* W83 Reduces Gingipain Activity and Alters Trafficking in and Response by Host Cells

**DOI:** 10.1371/journal.pone.0074230

**Published:** 2013-09-12

**Authors:** Leticia Reyes, Eileen Eiler-McManis, Paulo H. Rodrigues, Amandeep S. Chadda, Shannon M. Wallet, Myriam Bélanger, Amanda G. Barrett, Sophie Alvarez, Debra Akin, William A. Dunn, Ann Progulske-Fox

**Affiliations:** 1 Department of Oral Biology, College of Dentistry and Center for Molecular Microbiology, Gainesville, Florida, United States of America; 2 Department of Periodontology, College of Dentistry, University of Florida, Gainesville, Florida, United States of America; 3 Donald Danforth Plant Science Center, proteomics & mass spectrometry Core, St. Louis, Missouri, United States of America; 4 Department of Anatomy and Cell Biology, College of Medicine, University of Florida, Gainesville, Florida, United States of America; University of Illinois at Chicago College of Medicine, United States of America

## Abstract

*P. gingivalis* (*Pg*), a causative agent of chronic generalized periodontitis, has been implicated in promoting cardiovascular disease. Expression of lipoprotein gene *PG0717* of *Pg* strain W83 was found to be transiently upregulated during invasion of human coronary artery endothelial cells (HCAEC), suggesting this protein may be involved in virulence. We characterized the virulence phenotype of a *PG0717* deletion mutant of pg W83. There were no differences in the ability of W83Δ717 to adhere and invade HCAEC. However, the increased proportion of internalized W83 at 24 hours post-inoculation was not observed with W83∆717. Deletion of PG0717 also impaired the ability of W83 to usurp the autophagic pathway in HCAEC and to induce autophagy in Saos-2 sarcoma cells. HCAEC infected with W83Δ717 also secreted significantly greater amounts of MCP-1, IL-8, IL-6, GM-CSF, and soluble ICAM-1, VCAM-1, and E-selectin when compared to W83. Further characterization of W83Δ717 revealed that neither capsule nor lipid A structure was affected by deletion of PG0717. Interestingly, the activity of both arginine (Rgp) and lysine (Kgp) gingipains was reduced in whole-cell extracts and culture supernatant of W83Δ717. RT-PCR revealed a corresponding decrease in transcription of *rgpB* but not *rgpA* or *kgp*. Quantitative proteome studies of the two strains revealed that both RgpA and RgpB, along with putative virulence factors peptidylarginine deiminase and Clp protease were significantly decreased in the W83Δ717. Our results suggest that PG0717 has pleiotropic effects on W83 that affect microbial induced manipulation of host responses important for microbial clearance and infection control.

## Introduction

The Gram negative anaerobic bacterium *Porphyromonas gingivalis* is a predominant periodontal pathogen that has also been implicated in cardiovascular disease [1-3]. Genotyping of natural *P. gingivalis* populations reveals that the microbe has a high degree of genetic diversity, which may account for the wide range of virulence phenotypes associated with this organism [4,5]. Several comparative genomic approaches have been used to identify novel virulence genes of *P. gingivalis* [4,6,7]. These studies have identified multiple insertion sequences, hypothetical genes, and functionally assigned genes in the pathogenic W83 strain that are altered or missing in the genome of the less virulent strain 33277 [7,8]. *PG0717* is one of the hypothetical lipoprotein genes of W83 that is truncated in strain 33277 [7], and is also highly divergent among various *P. gingivalis* strains according to micro-array based comparative genomic hybridization analysis [6].

Although the biological function of *PG0717* is unknown, it has been annotated as a putative lipoprotein predicted to reside within the periplasmic space. We have confirmed that PG0717 is in the same operon with PG0718 (Figure **S1B** **, S1C and** Table **S1** ****in File **S1**), which is also predicted to be a periplasmic protein. *In silico* analysis with STRING [9] indicates that *PG0717* homologs and homologs of its neighbors *PG0718*, *PG0719*, and *PG720* are conserved within the order *Bacteroidales*. Interestingly, *PG0717* is predicted to interact with *PG0719* and *PG720*, which form a two-component histidine kinase signaling system. Two-component signal transduction systems regulate the expression of several bacterial genes in response to environmental and intracellular stimuli. *P. gingivalis* has several two-component sensor histidine kinase systems, which have been shown to enhance virulence by regulating the processing or expression of various virulence factors including major fimbriae [10], biofilm production [11], and the maturation and proper localization of gingipains [12]. Therefore, we hypothesized that PG0717 may modulate the virulence of W83 through a similar mechanism, namely, regulation of virulence factor expression or processing.

Of the proteases that *P. gingivalis* produces, the most noteworthy are a set of cysteine proteases referred to as gingipains. These molecules occur as both cell-associated and secreted forms [13-15]. One type of gingipain cleaves at lysine residues (lysine gingipain; Kgp), whereas two other proteases cleave proteins at arginine residues (arginine gingipains A and B; RgpA and RgpB) [15]. The gingipains share extensive amino acid sequence homology with each other and with the major hemagglutinin HagA. These molecules, and a number of others, share a C-terminal domain that is thought to be critical to their transport through the outer membrane via a unique transport system and attachment to the outer membrane [16-19].

In addition to gingipains, other surface entities are known to affect the virulence of *P. gingivalis*. The lipid A moiety of the bacterial lipopolysaccharide of *P. gingivalis* has been reported to influence the innate immune response, and thereby cytokine production, by its effect on Toll-like receptors [20-22]. Alterations in the structure of lipid A, including number of attached acyl and phosphate groups, can change the bacterial interaction with host cells from merely immune-evasive to actively immune-suppressing [20-22]. The capsular polysaccharide, which is not found on all strains of *P. gingivalis* [23] has been demonstrated to both alter cytokine production in cultured host cells [24,25] and influence the ability of the bacteria to disseminate *in vivo* [25,26]

The role of PG0717 as a potential virulence factor has not been determined. However, previous observations in our laboratory suggest that PG0717 may be involved in early host/pathogen interactions. Specifically, we have observed that expression of *PG0717* in W83 is significantly up-regulated during the first hour of invasion in human coronary artery endothelial cells (HCAEC) (unpublished data, Figure **S1A** in File **S1**). Therefore, in order to determine the pathogenic potential of PG0717, we constructed an isogenic mutant in W83 and assessed its effects on HCAEC. Deletion of *PG0717* produced a pleiotropic mutant with an altered virulence phenotype. W83∆717 lost the ability to manipulate the autophagic pathway during invasion of HCAEC. Further, W83∆717 infection of HCAEC elicited a more pronounced inflammatory response in these cells than its wild type counterpart. HCAEC responses to W83Δ717 could not be attributed to the capsule or lipid A structure of this mutant since it did not exhibit any alterations in these structures. However, W83Δ717 displayed a significant reduction in both Kgp and total Rgp gingipains activity. This coincided with decreased RgpA and RgpB protein levels in the proteome profile of W83Δ717. Quantitative proteome profiling of W83Δ717 also revealed decreased protein levels of other putative virulence factors including peptidylarginine deiminase and Clp protease.

## Results

### Deletion of *PG0717* Does Not Impact *P. gingivalis* Adherence to or Invasion of HCAEC but Reduces Resistance to Intracellular Killing

Details of the preliminary characterization of the *PG0717* deletion mutant, including confirmatory Northern blot analysis, are presented in File **S1** (Figure **S1B** **-S1E;** Table **S1**). A comparison of the growth curves of the two strains under standard broth culture conditions revealed no differences in the growth patterns over 21 h (Figure **S1F** ****in File **S1**), indicating that there is no inherent defect in growth of this mutant. The ability of W83Δ717 to adhere to and invade host cells was evaluated in HCAEC in order to determine if *PG0717* facilitated microbial invasion and survival in endothelial cells. Deletion of *PG0717* did not have a significant effect on the ability of *P. gingivalis* to adhere to HCAEC. The mean ± SD log CFU of *P. gingivalis* that adhered to HCAEC was 5.44 ± 0.13 for W83 and 5.57 ± 0.01for W83Δ717.

**Figure 1:**
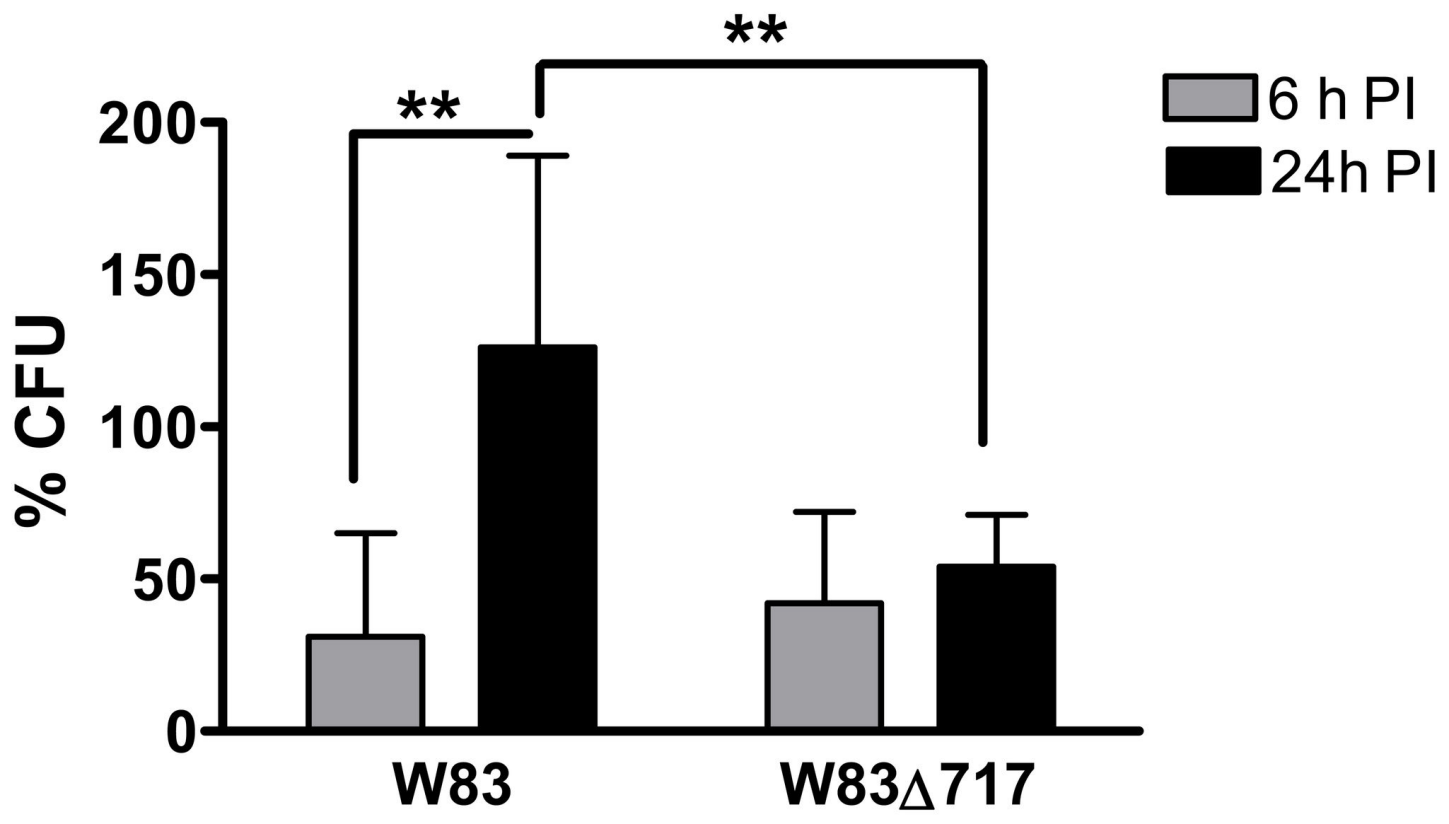
Intracellular persistence of *P. gingivalis* within HCAEC. Values represent the mean percent ± SD (*n* = 6) of invaded bacteria enumerated at 6 and 24 h PI. Percent values were determined by dividing the CFU obtained from 6 and 24 h infections by the average CFU obtained at time zero (2.5 h PI). **Values were significantly different as determined by Student’s *t*-test (*P* < 0.02).

Invasion assays were performed under constant antibiotic pressure so that cultures reflect intracellular bacteria isolated from HCAEC. *P. gingivalis* was not cultured from any supernatants from any invasion assays, thus confirming the efficacy of the antibiotic treatment. There were no significant differences in the ability of W83 and W83∆717 to invade HCAEC. The mean ± SD log CFU (mean ± SD % of inoculum) cultured from cell lysates after 2.5 hours of infection were 6.0 ± 0.5 (12 ±12%) for W83 and 6.4 ± 0.4 (14 ± 7%) for W83Δ717. The log CFU (% of inoculum) cultured from HCAEC lysates at 6 hours post-inoculation (PI) were 5.8 ± 0.6 (7 ± 8%) for W83 and 6.1 ± 0.5 (4 ± 4%) for W83Δ717. At 24 hours PI, the log CFU (% of inoculum) from W83 infected cells was 6.0 ± 0.2 (8 ± 5%) compared to 6.1 ± 0.3 (5 ± 5%) from W83Δ717 infected cells. In order to adjust for any variations in the ability of W83 and W83Δ717 to persist in HCAEC, we normalized the number of internalized bacteria at 6 and 24 hours PI by dividing the CFU of bacteria enumerated at these time points by the CFU of 2.5 hour PI cultures, which we designated as time zero (Figure **1**). There was no difference in the proportion of W83 and W83∆717 that were enumerated from 6 hour PI lysates. However, as the duration of infection progressed, the proportion of internalized W83 retrieved from 24 h lysates was significantly greater than the proportion of internalized W83 and W83∆717 isolated at 6 hours PI. Further, the proportion of W83 obtained from 24 hour cultures was significantly greater than the proportion of W83∆717 obtained at that same time point. These results suggest that deletion of PG0717 either reduces the resistance of W83 to intracellular killing and/or impairs the ability of W83 to replicate within the host cell.

### Deletion of *PG0717* Alters Intracellular Trafficking and Induction of Autophagy by W83

**Figure 2:**
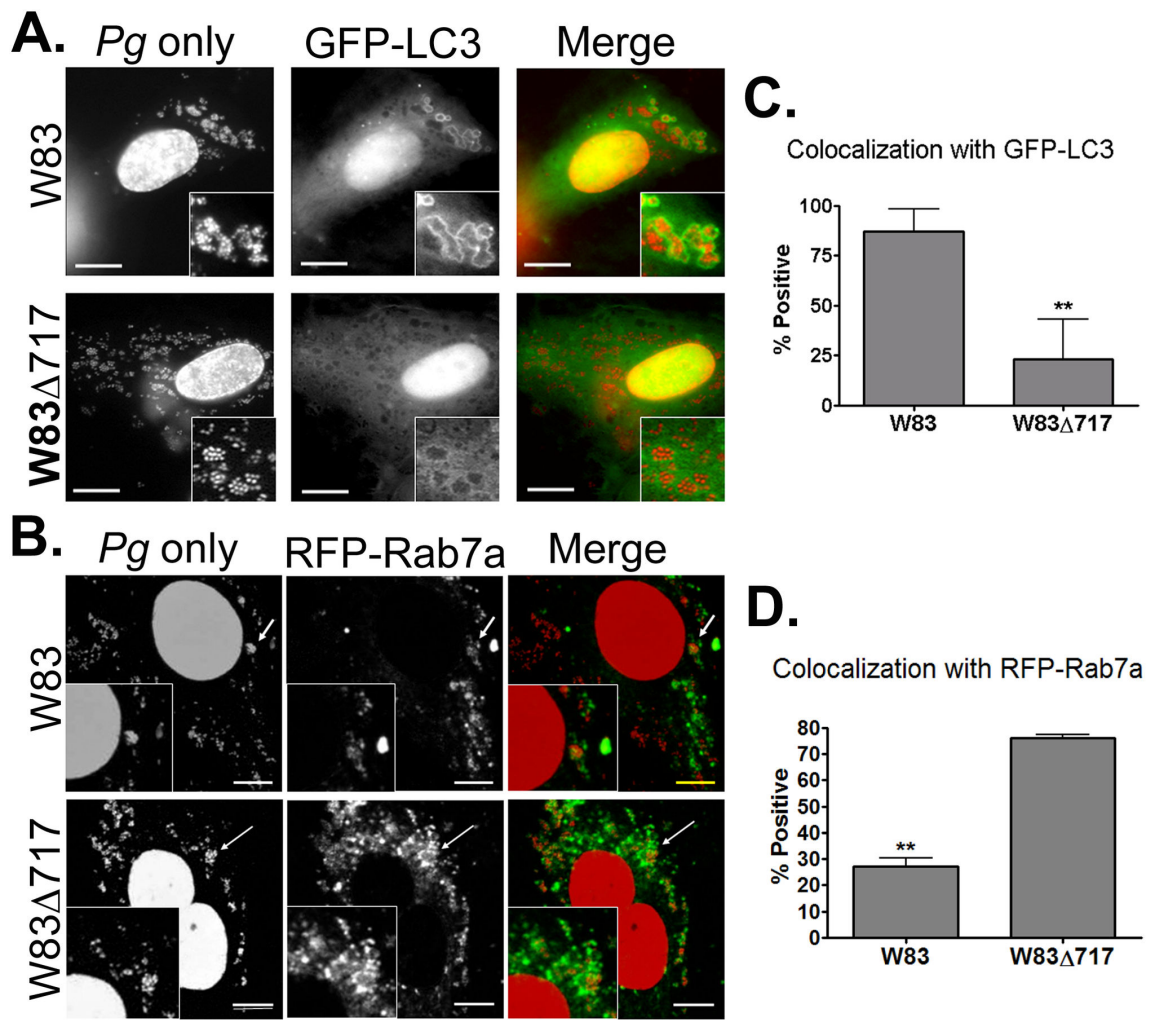
Intracellular trafficking of *P. gingivalis* in HCAEC. A) Colocalization of W83 and W83Δ717 (red) with GFP-LC3 (green) at 6 hours post-inoculation. B) Colocalization of W83 and W83Δ717 (red) with RFP-Rab7a (pseudocolored green) at 6 hours post-inoculation. Scale bar is equivalent to 10 µm. C) Mean percent ± SD (*n* = 3) of *P. gingivalis* present within GFP-LC3 positive vacuoles. D) Mean percent ± SD (*n* = 3) of *P. gingivalis* present within RFP-Rab7a positive vacuoles. Values were obtained from 3 independent experiments performed under constant antibiotic pressure. **Values were significantly different as determined by Student’s *t*-test (*P* < 0.01).

We have previously shown that *P. gingivalis* strain 381 activates autophagy in HCAEC whereby the autophagosome provides a replicative niche for the microbe within these host cells during invasion [27]. Strain W83 also primarily traffics through the autophagic pathway during invasion of HCAEC. However, unlike *P. gingivalis* 381, internalized W83 can survive within LAMP-1 positive endosomes following inhibition of autophagy [28]. In order to determine if deletion of *PG0717* altered the intracellular trafficking of W83 (Figure **2A-D**), we quantified the number of internalized bacteria within endosomes or autophagosomes of HCAEC that were transduced with early endosome specific marker RFP-Rab 5, autophagosome specific GFP-LC3, or late endosome specific RFP-Rab7a. *P. gingivalis*-infected cells were evaluated at the same PI points that were used in the above invasion assays and in previous studies [28]. Transduction controls for expression vectors can be viewed in File **S2**, Figures **S2A** and **S2B**.

There were no significant differences in the proportion of internalized W83 or W83∆717 within Rab5-positive vacuoles (Figure **S2C** ****in File **S2**). At 2.5 hours PI, 54 ± 6% of internalized W83 and 50 ± 4% of W83∆717 colocalized with Rab5. However, as quantified in Figure **2C and D**, at 6 hours PI, 83 ± 12% of internalized W83 were found within GFP-LC3-positive vacuoles (Figure **2C**), and only 27 ± 3% of W83 were found in Rab7a positive vacuoles (Figure **2D**). In contrast, only 23 ± 20% of internalized W83∆717 were found within autophagosomes (Figure **2C**), and 76 ± 1% were found in late endosomes (Figure **2D**).

**Figure 3:**
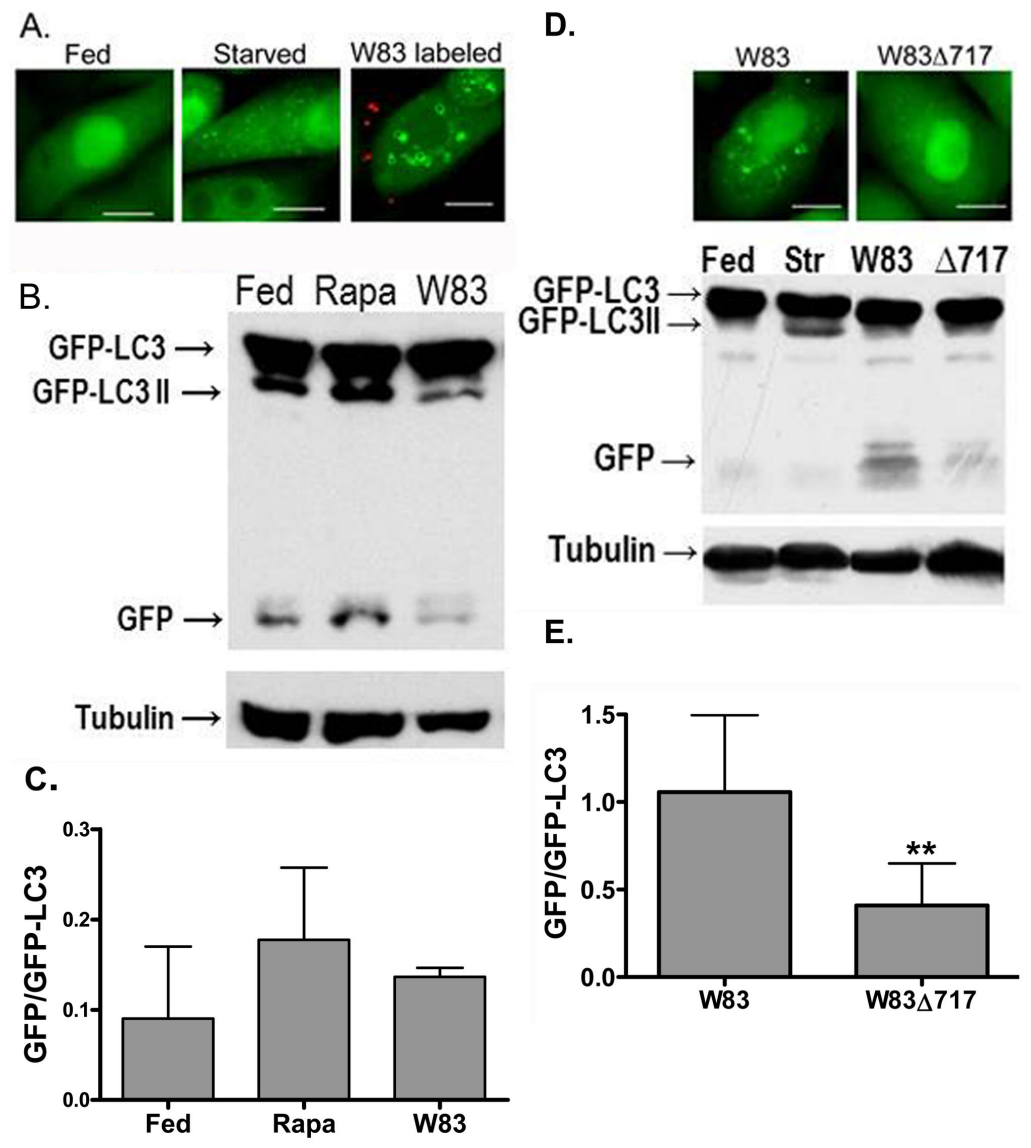
Induction of autophagy in Saos-2 cells. Saos-2 cells stably expressing GFP-LC3 were incubated in the presence and absence of *P. gingivalis*. After one hour, the cellular distribution of GFP-LC3 was visualized by fluorescence microscopy and its degradation assessed by Western blots. (A) Representative microscopic images of Saos-2 GFP-LC3 cells after one hour of starvation or co-culture with *P. gingivalis*. *P. gingivalis* were pre-labeled with Texas red before inoculation of Saos-2 cells. Scale bar is equivalent to 10 µm. (B) Saos-2 cell lysates were collected after 1 hour of fed conditions, rapamycin treatment (500 nM; “Rapa”), or incubation with W83. Tubulin was used as a loading control. A representative blot is shown. (C) Densitometric analysis of two Western blots including the blot shown in Figure 2B. (D) Representative microscopic images of Saos-2 cells after one hour of co-culture with W83 or W83Δ717 are shown in the upper segment; scale bar is equivalent to 10 µm. In the lower segment, Saos-2 cell lysates were collected after 1 hour of fed or nutrient-deprived (“Str”) conditions or after a one-hour incubation with W83 or W83Δ717 (“Δ717”). Tubulin was used as a loading control. (E) The degradative removal of GFP was significantly higher in cells exposed to W83 than in those incubated with W83Δ717. Values represent the mean ± SD of 4 independent experiments. **Ratios were significantly different as determined by unpaired Student’s *t*-test (*P* < 0.04).

Next, we assessed whether internalization of *P. gingivalis* by the host cell was necessary to elicit the autophagic response as measured by the formation of LC3-positive vacuoles. Accordingly, we utilized a cell line that, in our hands, does not internalize *P. gingivalis* W83 (Figure **3A**), Saos-2, that has been transfected to stably express GFP-LC3 as a reporter for the induction of autophagy. In this manner, the contribution of factors secreted by extracellular *P. gingivalis* to the induction of autophagy could be evaluated independently of bacterial internalization. Conjugation of LC3-I to phosphatidylethanolamine at the C-terminus converts the protein to LC3-II, which can be found tightly bound to the membrane of the autophagosome and serves as a marker for these vacuoles [29,30]. However, although formation of LC3-II correlates with autophagosome formation [30], LC3-II by itself is not a reliable measure of autophagic flux since LC3-II can be recycled back into the cytosol as LC3-I instead of being degraded. Accordingly, both microscopy to detect LC3-II-containing vacuoles (Figure **3A** and Figure **3D**) and Western blot analysis of cell lysates to determine the proteolytic generation of GFP from GFP-LC3, which is indicative of LC3 degradation and thus autophagic flux [31] (Figure **3B** and Figure **3D**, corresponding densitometric analyses in Figure 3C and 3E), were employed.

After one hour of starvation, a condition which induces autophagy, GFP-LC3 positive vacuoles were detected within uninfected Saos-2 cells, whereas GFP-LC3 positive punctae were absent or few in number in fed uninfected cells (Figure **3A**). Interestingly, large GFP-LC3 positive vacuoles were present in fed Saos-2 cells that were co-cultured with W83 (Figure **3A** and Figure **3D**), suggesting autophagy was activated. Furthermore, in parallel experiments in which the Saos-2 cells were co-cultured with rapamycin, a known activator of autophagy [32], or with W83, we found that co-culture with the bacterial cells resulted in the proteolytic release of GFP, as was observed with rapamycin treatment (Figure **3B** - Figure **3D**), indicating that the LC3 was being degraded.

In contrast, the number of GFP-LC3 positive vacuoles in Saos-2 cells co-cultured with W83Δ717 did not exceed background levels (as observed in fed uninfected cells similar to that shown in Figure 3A), indicating this strain did not activate autophagy (Figure **3D**). These findings were substantiated in the Western blots of Saos-2 cells co-cultured with W83Δ717, wherein the proteolytic release of GFP form GFP-LC3 was minimal (Figure **3D**, right 2 lanes of lower segment; quantified in Figure **3E**). The data are consistent with *PG0717* being required for the activation of autophagy in Saos-2 cell lines.

### PG0717 Is Involved in Early Activation of Endothelial Inflammatory Responses

**Figure 4:**
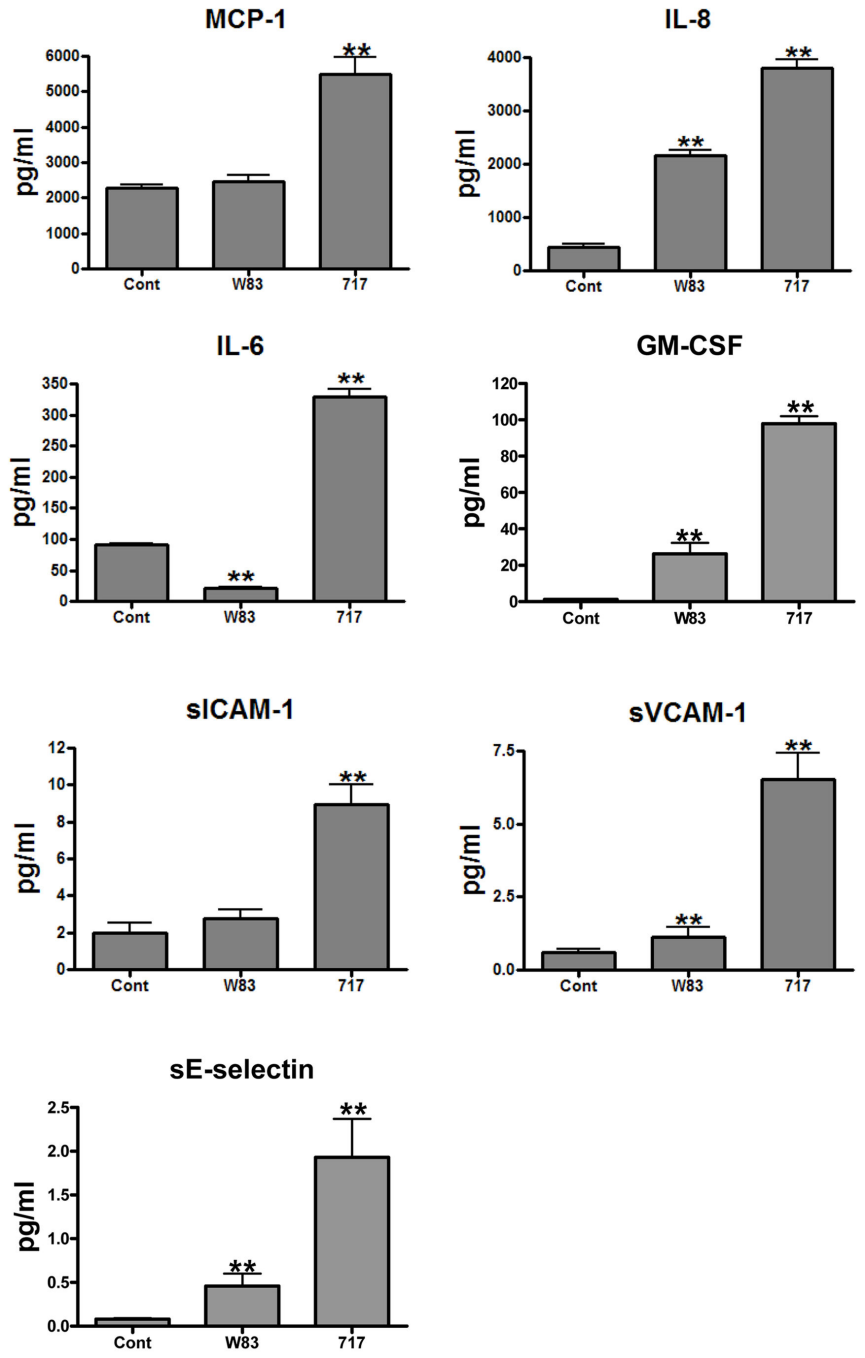
Inflammatory mediator profile of uninfected and *P. gingivalis* infected HCAEC. HCAEC were infected with 10^7^ bacteria (MOI of 100). Cell culture supernatants were collected at 24 hours PI and analyzed with Milliplex detection kits. Values represent the mean ± SD concentration from 3 independent experiments (*n* = 3). **Indicates group means that were significantly different by ANOVA followed by Fisher’s multiple comparison test (*P* < 0.001).

Infection of endothelial cells with *P. gingivalis* will induce the expression or secretion of pro-inflammatory cytokines/chemokines and cell adhesion molecules [28,33,34], which is an early indicator of endothelial activation [35,36]. Therefore, cell culture supernatants collected at 24 h post infection were analyzed for the presence of MCP-1, IL-8, IL-6, GM-CSF, and soluble cell adhesion molecules (ICAM-1, VCAM-1, and E-selectin) (Figure **4**). HCAEC cells infected with W83 secreted higher amounts of IL-8, GM-CSF, sVCAM-1, and sE-selectin than uninfected cells (*P* < 0.01). In contrast, cells that were infected with W83∆717 expressed higher levels of IL-8, GM-CSF, sVCAM-1, and sE-selectin when compared to those infected with W83. Unlike W83 infected cells, W83∆717 infected cells expressed elevated levels of MCP-1, sICAM-1, and IL-6, when compared to uninfected controls (*P* < 0.001).

### The Different Host Cell Response to PG0717 vs. W83 Is Not Due to Alterations in Lipid A or Capsule

The lipid A moiety of *P. gingivalis* lipopolysaccharide has been reported to influence host cell cytokine and chemokine responses through manipulation of Toll-like receptor (TLR) mediated signaling [21,22]. Alterations in TLR signaling can also have an effect on host cell autophagic responses [37]. In addition, loss of capsule expression in *P. gingivalis* can result in enhanced host inflammatory responses during infection [24,25]. Therefore, we assessed whether the observed increase in inflammatory cytokines, the impaired ability of the deletion mutant to induce autophagy, or both, could be attributed to alterations in these cell surface components. Bacterial lipopolysaccharide was purified at equivalent yields and submitted for qualitative MALDI-TOF analysis of the lipid A structure. As shown in File **S1**, Figure **S1G**, there was no appreciable difference in the spectra between the lipid A molecules from the deletion mutant and W83; both the 1448 and 1688 species are present in the lipid A samples from both strains. Electron microscopic analysis of bacterial cells stained with ruthenium red revealed no significant differences in the appearance or thickness of the capsular polysaccharide between W83 and W83Δ717 (File **S1**, Figure **S1H**).

### Gingipain Activity and Expression

Gingipain-mediated destruction of cytokines has been implicated as contributing to dampening of the immune response by vascular endothelial cells when infected with *P. gingivalis* [38]. To address the possibility that the increase in cytokine production observed in W83Δ717-infected cells was owing to diminished gingipain activity by the mutant, *in vitro* measurements of gingipain activity were carried out. Activity curves can be viewed in File **S1**, Figure **S1I**. Both cell-associated and secreted gingipain activities were reduced, 14% of the total secreted arginine gingipain (RgpA plus RgpB) activity and more than 70% of the secreted Kgp activity in W83Δ717 (Table **1**)**.**

**Table 1:** Gingipain activity associated with whole cells and culture supernatants of deletion mutant W83Δ717 vs. those of W83.

		Enzyme Activity (microunits)			
Strain	Rgp Supernatant		Rgp Pellet	Kgp Supernatant	Kgp Pellet
W83	0.217		4.35	0.079	1.09
W83Δ717	0.186 (85.7%)		3.04 (70.0%)	0.023 (29.4%)	0.870 (80.0%)

The activity is the amount (× 10^6^ pmol) of *p*-nitroaniline liberated min^- 1^ µl^- 1^ of reaction volume. In the W83Δ717 row, the percentage of the activity found in W83 is shown in parentheses. *P* < 0.0001 for all enzyme activities in W83Δ717 compared to those in W83.

In order to determine if reduced gingipain activity could be attributed to changes in the transcription levels of *rgpA*, *rgpB*, and *kgp*, expression levels of these genes was assessed by RT-PCR. To account for the possibility that the decrease in gingipain activity was owing to alterations in the expression of genes involved in gingipain processing, secretion, or anchoring, transcription levels of *vimE* and *porT* [39,40] were also measured. RT-PCR revealed that expression of one of the arginine gingipain genes, *rgpB*, was decreased in the deletion mutant compared to W83 (Table **2**). However, the expression of the other genes encoding for gingipain enzymes (*rgpA* and *kgp*) was unaffected in the mutant. Furthermore, deletion of *PG0717* did not impact the expression of either *porT* or *vimE*, suggesting that the decrease in gingipain activity was not attributable to a general defect in expression of genes associated with gingipain transport or processing.

**Table 2:** Expression of gingipain genes as measured by RT-PCR.

	Relative Gene Expression (2^-^^(ΔCT)^)^*a*^	
Gene name	W83	W83Δ717
*rgpA*	2.542 ± 0.525	2.402 ± 0.490
*rgpB*	2.040 ± 0.435	1.186 ± 0.142^*b*^
*kgp*	0.081 ± 0.032	0.075 ± 0.013
*vimE* ^*c*^	0.313 ± 0.063	0.376 ± 0.085
*porT* ^*d*^	0.146 ± 0.030	0.115 ± 0.014

^a^Mean ± SD of quintuplicate samples.

^b^ *P* < 0.005 compared to expression of this gene in W83. All other *P*-values were > 0.05.

^c^ Included for reference: VimE is involved in gingipain processing, secretion, or anchoring [39].

^d^ Included for reference: PorT is involved in the gingipain secretion system [40].

### Labeling Quantitative Proteomics

The amine-specific peptide-based labeling system (iTRAQ®) has been used extensively as a means of identifying microbial cellular processes that are altered by mutation [41-44]. Therefore, we used this approach to determine if deletion of *PG0717* affected processes that may be particularly relevant to host/pathogen interactions. With this in mind, W83 and W83∆717 were cultivated under the same conditions used to prepare inoculates for infection of HCAEC.

The LC-MS/MS experiment identified 355 proteins with 95% confidence. Detailed lists of identified proteins, their assigned biological function, and their relative abundance are found in Tables **S3A** and **S3B** in File **S3**. Of the 355 proteins that were identified, 122 proteins (Table **S3A**, File **S3**) exhibited a significant change in abundance (*P* < 0.05) as a result of *PG0717* deletion. Most of these proteins are associated with a variety of biological processes including metabolism, genetic processes, transport, stress response, protein biosynthesis, and protein processing. An equivalent proportion of proteins within these categories did not change in W83Δ717 (Table **S3B**, File **S3**).

Notable features that were detected in this analysis include proteins involved cell wall biogenesis and alterations in putative virulence factors. For example, none of the proteins involved in peptidoglycan synthesis were affected by deletion of *PG0717* (Table **S3B**, File **S3**). However, all putative virulence proteins [45-49] that were detected by LC-MS/MS were significantly perturbed in W83Δ717 protein fractions (Table **S3A**, File **S3**). Specifically, hemagglutinins (Hag) HagA and HagC, which have been shown to facilitate attachment and invasion [45,50,51], were increased in the W83Δ717 protein fractions. In contrast, gingipain proteins, RgpA (HagE) and RgpB were decreased in W83Δ717. Other proteins that were decreased in W83Δ717 include Clp protease, which is implicated in facilitating evasion of the endosome/lysosome pathway [52], and peptidylarginine deiminase, which may enhance colonization and virulence of *P. gingivalis* by inactivating antimicrobial peptides [53,54]. In this experiment, lysine gingipain (Kgp) was not identified.

## Discussion

Important features of *P. gingivalis* mediated disease include the ability of the microbe to attach and invade host cells, disseminate through host tissues, and subvert host immunological surveillance and defense mechanisms [55]. These features are largely executed through well characterized virulence factors such as cysteine proteases (gingipains), fimbriae, lipopolysaccharide, capsule, and hemagglutinins that exert their effects through direct interactions with the host [49,55]. In addition, *P. gingivalis* can modulate its pathogenicity by regulating the expression or processing of its virulence properties. Examples of this mode of regulation include VimA [56] and the GppX two-component sensor kinase system, which regulate the production or processing of gingipains and/or fimbriae [10,12]. Our studies confirmed that PG0717 indirectly enhances virulence of W83 through pleiotropic effects on *P. gingivalis* virulence properties including Rgp and Kgp gingipains, which, in turn, impact the ability of the organism to manipulate host responses involved in microbial clearance and subsequent control of the infection.

We evaluated the response of HCAEC to W83∆717 since prior studies by our group indicated that the expression of *PG0717* was enhanced during adherence and early invasion of these cells (File **S1**, Figure **S1A**). Interestingly, deletion of *PG0717* did not have a significant effect on the early stages of invasion (adherence and cell entry). However, deletion of PG0717 impacted the pattern of W83 persistence within HCAEC. Namely, the number of internalized W83 increased with duration of infection whereas the numbers of internalized W83∆717 remained essentially the same. This change coincided with a loss in the ability of W83∆717 to manipulate the autophagic pathway. Both *P. gingivalis* strain 381 and W83 can perturb the autophagic pathway during invasion of HCAEC, avoiding lysosomal degradation and utilizing the autophagosome as a replicative niche whereby the microbe can benefit from the peptide rich microenvironment of the autophagosome [27,28,57]. The shuttling of W83∆717 into the endosome/lysosome pathway coupled with the static number of internalized W83∆717 over a 24 hour period reaffirms that *P. gingivalis* benefits from manipulation of the autophagic response and that PG0717 plays a role in facilitating that process.

Previous studies by Dorn et al. provided evidence that *P. gingivalis* subverts autophagy by delaying the fusion of the late autophagosome with the lysosome [27,57]. However, these studies were unable to establish whether *P. gingivalis* actively induced autophagy in host cells, or if the microbe was adapting to a defense response initiated by the host during microbial invasion [58]. The ability of W83 to induce autophagy in Saos-2 cells without invading these cells, coupled with the inability of W83Δ717 to do so, suggests that *P. gingivalis* plays an active role in initiating the autophagic response and this is mediated by secretion or shedding of pro-autophagic factors prior to direct contact with the host cell.

It has been proposed that secretion or shedding of microbial proteinases within the autophagosome is one mechanism by which *P. gingivalis* subverts the autophagic response [27]. This theory is based on evidence of increased proteolysis of long-lived endogenous proteins of HCAEC infected with *P. gingivalis*, and microscopic evidence of *P. gingivalis* shedding secretory vesicles presumed to be rich in proteinases while the bacterium is residing within late autophagosomes [57]. Our experiments involving W83∆717, which exhibited decreased gingipain activity and failed to induce autophagy in both HCAEC and Saos-2 cells, also provide support for this theory.

A distinguishing feature between 381 and W83 is that 381 depends on the autophagic pathway for its intracellular survival, whereas W83 does not in that it can survive within the HCAEC endosome pathway if the autophagic response is inhibited [28]. This feature was reaffirmed by our trafficking studies with autophagy deficient W83Δ717 in that it demonstrated that W83Δ717, which primarily trafficked through the endosome pathway, was equally capable of surviving within HCAEC. In *E. coli*, expression of K1 capsule can delay endosome/lysosome fusion during invasion of human brain microvascular endothelial cells [59]. Thus, it is possible that K1 capsule expression by both W83 and W83Δ717 may have a similar effect during invasion of HCAEC. Yamatake et al. have previously demonstrated that gingipains enhance the survival of unencapsulated *P. gingivalis* within endolysosomes, thus, it is also possible that W83Δ717 retains sufficient gingipain function to protect it from lysosomal attack [60].

Deletion of *PG0717* also affected host cell cytokine and cell adhesion expression that could impact the progression or severity of disease. For example, microbial disruption of host cytokine production and regulation of cell adhesion molecule expression during infection can serve as a means of evading innate defenses that promote leukocyte chemotaxis and diapedesis into the affected tissue site. Previously, we noted that HCAEC infected with W83 exhibited a reduced pro-inflammatory response when compared to *P. gingivalis* strains 381 and 33277 [28]. Similar to our previous findings, here W83 infected cells exhibited an attenuated pro-inflammatory response but deletion of *PG0717* resulted in an increased pro-inflammatory response. This effect could not be attributed to LPS structure or capsule composition since neither of these components was altered in W83Δ717. However, the significant decrease in both Rgp and Kgp activity of W83Δ717 may, in part, account for the pro-inflammatory effects we observed in HCAEC infected with this mutant. For example, infection of endothelial cells with a Kgp deficient mutant results in an increase in both IL-8 transcription and protein expression relative to cells exposed to the wild-type strain of *P. gingivalis* [33]. Madrigal et al. have recently demonstrated that Kgp gingipain disrupts intracellular kinases that act as downstream effectors of Toll-like receptor (TLR), TNF-α, and Nod-like receptor (NLR) mediated production of cytokines [61]. Cytokine/chemokine networks can also be disrupted by gingipain mediated proteolysis [62-64].

We used quantitative proteome profiling as a method to screen for any additional phenotypic changes that occurred with deletion of *PG0717*. In order to best approximate the *P. gingivalis* W83 and W83Δ717 phenotypes that interacted with host cells, protein extracts were prepared from bacteria that were grown under the same conditions as the inoculates used in HCAEC infection studies. Interestingly, all virulence associated proteins that were detected by this analysis were found to be significantly changed in W83Δ717, indicating that PG0717 may affect multiple virulence properties of W83.

The decreased levels of RgpA and RgpB in the protein fractions of W83Δ717 were consistent with decreased Rgp activity of this mutant. However, the mechanism by which PG0717 affects Rgp activity is not clear. Whereas *rgpB* transcripts were significantly decreased in W83Δ717, the same effect was not observed in *rgpA*. Kgp was not detected by iTRAQ® analysis, which may be due to extremely low protein levels as suggested by the low amount of *kgp* gene transcript levels in both W83 and W83Δ717 RT-PCR analysis. Other noteworthy changes within the proteome profile were the decrease in ATP-dependent Clp protease and peptidylarginine deiminase in the protein fraction of W83Δ717. Clp protease has been shown to modulate *P. gingivalis* trafficking within host cells [52]. Relevant to W83Δ717, is that *clpP* deletion mutants of *P. gingivalis* 33277 primarily traffic into lysosomes during invasion of gingival epithelial cells [52]. Thus, ClpP may facilitate evasion of the endosome/lysosome pathway by W83 during invasion of HCAEC. To date, *P. gingivalis* is the only known bacterium that expresses peptidylarginine deiminase (PPAD) [53]. PPAD may promote virulence properties of *P. gingivalis* by enhancing *in vivo* colonization of the host through inactivation of antimicrobial peptides [53] and host derived peptides that inhibit *P. gingivalis* hemagglutinating activity [54].

It must be acknowledged that the pleiotropic effects that we observed in W83∆717 may be the result of a disrupted protein complex such as a two-component sensor histidine kinase signaling system. The gene context of *PG0717*, specifically, its orientation with *PG0718, PG0719*, and *PG0720* is conserved among several members of the order *Bacteroidales* [9] implying that their gene products may be functionally associated. For example, in W83, *PG0719* and *PG0720* sequences resemble the basic elements of a two- component system: the histidine kinase (PG0719) and the DNA-binding response regulator (PG0720) [65]. It is conceivable that PG0717 and PG0718 act as auxiliary regulatory proteins for PG0719/PG720 since bacterial two-component signaling systems are often regulated in this manner [66].

Our results indicate that PG0717 plays a role in microbial induced manipulation of host responses important for microbial clearance and infection control. Deletion of *PG071*7 produced a mutant that lost the capacity to manipulate the host autophagic response and failed to attenuate the production of pro-inflammatory mediators that trigger antimicrobial responses. In addition, perturbed HCAEC responses to W83Δ717 coincided with alterations in several putative *P. gingivalis* virulence factors including Rgp and Kgp gingipains, Clp protease, and peptidylarginine deiminase. The pleiotropic effects of PG0717 suggest that this protein may be involved in the regulation or processing of multiple virulence properties of *P. gingivalis*.

## Materials and Methods

### Bacterial Strains and Culture Conditions

Both W83 and W83Δ717 were maintained on blood agar plates (5% sheep blood, Quad-Five, Ryegate, MT, USA) supplemented with vitamin K_1_, hemin, yeast extract, and L-cysteine hydrochloride (sBAP) as set forth in Bélanger et al. [67]. Where required, gentamicin (50 µg/ml; Sigma-Aldrich, St. Louis, MO, USA) or erythromycin (10 µg/ml; Sigma-Aldrich) was added to sBAP. Tryptic soy broth supplemented as above (sTSB) [67], but without antibiotics, was employed for liquid cultures. Unless otherwise indicated, all cultures were incubated at 37°C in an anaerobic chamber (5% CO_2_, 10% H_2_, and 85% N_2_) (Coy Products, Ann Arbor, MI, USA), and all sTSB cultures were harvested at early stationary phase. Initial bacterial concentrations in all inoculates were determined by optical density readings performed at 550 nm. Bacterial suspensions were diluted in cell culture media to achieve an MOI of 100 for all infection experiments. The colony forming unit (CFU) of each inoculum was confirmed by culture.

### Mutant Construction

Primers for mutant construction, Northern blot analysis, and sequencing are listed in File **S1**, Table **S1**. W83Δ717 was created by allelic replacement using plasmid PR-UF1 and the protocols previously described [67]. Targeted disruption of the *PG0717* locus was confirmed by Northern blot and sequencing as shown in File **S1**, Figure **S1C** **- S1E**. For Northern blot, the probes were generated from unique 100-bp regions in the center of *PG0717* and *PG0718*. Probes were labeled with the BrightStar® Psoralen-Biotin labeling kit (Ambion Inc., Austin, TX, USA). RNA from *P. gingivalis* strains was extracted using Trizol according to the manufacturer’s instructions. DNA contamination was removed by digestion with DNase I, and samples were cleaned using the Invitrogen PureLink Mini Kit protocol for purifying RNA from liquid samples. RNA quality was assessed with a BioAnalyzer (Agilent Technologies, Inc., Santa Clara, CA, USA), and total RNA concentrations were determined with a NanoDrop system (Thermo Scientific, Rockford, IL, USA). Northern blot was performed using the NorthernMax formaldehyde-based system (Ambion); 10 µg of total RNA was loaded into each lane for the analysis. Northern blot images were captured with BioRad ChemiDoc XRS using Quantity One software.

### Growth Curves

Comparison of the growth of W83 and W83Δ717 was carried out in liquid cultures inoculated with an overnight (18-h) liquid culture of each organism at on OD_550_ of 0.10 ± 0.01. Growth was monitored spectrophotometrically at OD_550_ on a SmartSpec Plus spectrophotometer (Bio-Rad, Hercules, CA, USA) every 3 h for 21 h.

### Cell Lines and Culture Conditions

Human coronary artery endothelial cells (HCAEC) obtained from Lonza (Walkersville, MD, USA) were cultured in EBM®-2 plus SingleQuots® medium (Lonza), and maintained at 37°C/5% CO_2_. Only HCAEC that underwent less than nine passages were used for experiments. Sixteen hours before inoculation with bacteria, HCAEC were seeded at a concentration of 1 × 10^5^ cells per well on 12-well cell culture plates and maintained at 37°C/ 5% CO_2_. Saos-2 cells (American Type Culture Collection, Manassas, VA, USA) were maintained in Dulbecco’s Modified Eagle’s medium supplemented with 10% bovine growth serum (BGS, Thermo Scientific).

### HCAEC Adherence Invasion Studies

HCAEC were seeded at a concentration of 1 × 10^5^ cells per sample and maintained at 37°C/5% CO_2_ for 16 h before infection. For adherence assays, HCAEC were washed 3 times with antibiotic free EBM-2 after which both the bacterial preparation and HCAEC were chilled on ice for 15 min. Chilled HCAEC were then inoculated with *P. gingivalis* at an MOI of 100 and incubated at 4°C for 30 min without agitation. Next, HCAEC were washed twice with ice cold EBM-2. For invasion assays, a spin inoculation protocol was used in order to synchronize bacterial contact with host cells [68]. For continuous invasion studies, HCAEC were undisturbed until time of harvest. For invasion studies performed under antibiotic pressure, HCAEC cultures were washed 3 times to remove any remaining extracellular bacteria at 1.5 h post-inoculation (PI). Thereafter, cell cultures were maintained with EBM-2 media supplemented with 300 µg/ml gentamicin and 200 µg/ml metronidazole (Sigma-Aldrich) to kill any remaining extracellular bacteria. At the time of harvest, cell culture supernatants were cultured for the presence of live bacteria and stored at -80°C for cytokine analysis. In order to release intracellular bacteria, washed HCAEC cells were incubated in sterile distilled water for 20 min at 37°C/5% CO_2_. Cell lysates were serially diluted in sterile PBS, and the number of viable *P. gingivalis* organisms was determined by culture.

### *P. gingivalis* / HCAEC Vesicle Colocalization Studies

HCAEC were transduced with fluorescent protein-signal peptide fusion vectors used at an MOI of 10. Autophagosomes were tagged with green fluorescent protein (GFP)-light chain three (LC3) packaged in an adenovirus expression system (Welgen, Inc, Worcester, MA, USA). Early (Rab5) and late endosomes (Rab7a) were tagged with red fluorescent protein (RFP) packaged in a baculovirus system (*CellLight^®^ BacMam 2.0*, Life Technologies). After 48 h, cells were inoculated with *P. gingivalis* as described above. At 6 h post-inoculation, HCAEC were fixed with 4% paraformaldehyde dissolved in phosphate buffered saline (PBS). Fixed cells were washed three times with PBS before mounting with ProLong® Gold Antifade reagent with DAPI (Invitrogen™). HCAEC were visualized with an Olympus DSU-IX81 Spinning Disc Confocal microscope. Images were captured with Slidebook software (Olympus, Center Valley, PA). At least five images at 20X magnification were obtained from each sample. Final processing of images was performed with ImageJ software (US National Institutes of Health, Bethesda, MD).

### Autophagy Assessments

Saos-2 cells that stably express LC3 tagged with GFP were used to assess the ability of W83 and W83Δ717 to induce autophagy. Saos-2 cells were initially transfected with the pEGFP-mLC3 vector [30] using Lipofectamine 2000 (Invitrogen, Carlsbad, CA, USA) according to the protocol supplied by the manufacturer. The cells were grown under G418 selection (0.5 mg/ml) for a stable transfection and then cloned by limiting dilution; final sorting was carried out in the ICBR Core Facility for Flow Cytometry, University of Florida. The enriched transfected cells were maintained in Dulbecco’s Modified Eagle’s medium supplemented with G418 and 10% BGS. The day before inoculation with bacteria, the cells were seeded into 12-well cell culture plates at an approximate cell density of 2.0 × 10^5^ cells per well (microscopy) or 5.0 × 10^5^ cells per well (western blots) and maintained in antibiotic-free media. The Saos-2 GFP-LC3 cells were exposed to either standard DMEM with amino acids and BGS (“fed”), Krebs-Henseleit buffer without amino acids and BGS (“starved”), fed conditions with the addition of rapamycin (final concentration 500 nM, Sigma-Aldrich R-0395) or fed conditions with one of the bacterial suspensions at an MOI of 100, all at a volume of 1 ml per well, for 1 h at 37°C/5% CO_2_. The media was aspirated from the cells at 1 h post-inoculation and processed either for Western blot as set forth in the next paragraph or for microscopy by fixation with 4% paraformaldehyde dissolved in phosphate buffered saline (PBS). Fixed cells were washed three times with PBS before mounting with VectaShield® Mounting Medium with DAPI (Vector Labs, Burlingame, CA, USA). GFP-LC3 punctae within Saos-2 GFP-LC3 cells were visualized on a Leica DM IRBE microscope at 40X magnification using a GFP filter. At least 5 images per slide were randomly captured with OpenLab software (Improvisation). Final processing of images was carried out using ImageJ software (US National Institutes of Health, Bethesda, MD, USA).

For Western blots, 200 µl of Laemmli sample buffer containing protease inhibitor cocktail (Sigma-Aldrich) at a 1:100 dilution was added to each well. After freezing overnight, the samples were boiled for 5 min, run on a 10% polyacrylamide gel, and blotted onto a PVDF membrane for 1 hr using a semi-dry BioRad TransBlot blotter. After blocking with 5% nonfat dried milk in PBS with 0.1% Tween 20 (PBSTw) for 1 h at 25°C, the membrane was incubated with a mouse anti-GFP antibody (Sigma-Aldrich) at a 1:5,000 dilution for 18 h at 4°C. Horseradish peroxidase-conjugated goat anti-mouse IgG (Sigma-Aldrich) was employed at a 1:10,000 dilution in 2% milk in PBSTw for 2 h at 25°C. After several washes with PBSTw, the blots were transferred to a chemiluminescent solution and imaged on x-ray film.

Quantitation of the cleavage of GFP-LC3 was accomplished by scanning the film into a PC and analyzing the scanned image file using ImageJ software. For each lane, the bands corresponding to intact GFP-LC3 and cleaved GFP afforded distinct curves; the area under the curve was calculated for each band, and the ratios of the cleaved product to intact GFP-LC3 were calculated in Microsoft Excel (Microsoft, Redmond, WA).

### Detection of Cytokines, Chemokines, and Soluble Cell Adhesion Molecules

Culture supernatants that were harvested at 24 h post-inoculation with sterile media (control) or *P. gingivalis* were analyzed by Milliplex detection kits as previously described [28].

### Lipopolysaccharide Purification and Lipid A Extraction and Analysis

Bulk 24-h sTSB cultures of both W83 and W83Δ717 were grown from overnight starter cultures that were generated as for the growth curve studies. The bacteria were harvested at 6100 × *g* for 20 min at 4°C, washed once in ultrapure water, suspended in one-fifth the original volume of ultrapure water, and lyophilized. Purification of the bacterial lipopolysaccharide was carried out by the Tri-Reagent procedure of Yi and Hackett [69] as modified by Al-Qutub et al. [21]. Analysis of the lipid A structure was performed via matrix-associated desorption-time of flight mass spectrometry (MALDI-TOF/MS) as described in Guo et al. [70].

### Capsule Staining

Ultrastructural analysis of extracellular capsular material was carried out via ruthenium red staining of 48-hr cultures as described in detail elsewhere [71]. Specimens were post-fixed in 1% osmium tetroxide in 0.1 M sodium cacodylate, dehydrated and embedded in Epon 812 resin. Thin sections (60–80 nm) were cut and examined on a JEOL 100CX transmission electron microscope.

### Gingipain Assay

Measurement of the activity of the arginine (Rgp) and lysine (Kgp) gingipains was performed by a modification of the colorimetric procedure of James et al. [72]. Two milliliters of bacterial culture, OD_550_ = 1.0, were harvested by centrifugation at 5000×*g* for 5 min at 4°C. Subsequent handling of the bacterial suspensions, supernatants, and buffers was carried out on ice until the readings were taken. The supernatant was filtered and mixed 1:1 with reaction buffer (50 mM Tris hydrochloride, pH 7.5; 1 mM CaCl_2_; 5 mM cysteine). The cell pellet was suspended in 2.0 ml of reaction buffer, and 16 µl of this suspension was added to 184 µl of reaction buffer for each well. N_α_-benzoyl-DL-arginine *p*-nitroanilide hydrochloride (BAPNA) and *N*-tosyl-glycyl-L-prolyl-L-lysine 4-nitroanilide acetate salt (Z-GPK-pNA) (0.5 mM each; Sigma-Aldrich) were used as the substrate solutions for Rgp and Kgp, respectively. In quintuplicate for each cell pellet or supernatant, 100 µl of prepared suspension or supernatant was added to 100 µl of substrate solution in an ice-cold 96-well microtiter plate (Corning Costar, Tewksbury, MA, USA). The plate was then incubated at 37°C in a Wallac, Victor 3 microplate reader (PerkinElmer, Waltham, MA, USA) connected to a Windows 2000-based PC workstation; *A*_405_ readings were taken every minute for 2 h. The average and standard deviation were calculated in Microsoft Excel for each quintuplicate sample. The enzyme activity of each preparation was calculated from the derived *A*_405_ vs. time curves (File **S1**, Figure **S1I**) using a picomolar extinction coefficient (ε) of 9200 for the product, *p*-nitroaniline [73]. Statistical analysis of the differences in the *A*_405_ vs. time curves for each strain was carried out using used a linear mixed model in the SAS software program (Version 9.3) by the Biostatistical Consulting Laboratory, Department of Biostatistics, University of Florida (Gainesville, FL).

### Analysis of Gingipain Gene Expression

Bacterial cells grown under the same conditions employed for the gingipain enzyme assays were processed for quantitative RT-PCR. Briefly, total RNA was extracted from the cell pellet using Trizol® reagent. Expression of *rgpA*, *rgpB*, *kgp*, *vimE*, and *porT* were determined by employing primers that have been reported in the literature [39,40,74] with SYBR green detection (Qiagen). The PCR reactions were performed according to the manufacturer’s instructions on an iCycler-IQ, version 3.1 using with Optical System Software 3.1 (BioRad). Real-time PCR data was analyzed by the comparative threshold cycle (*C*_*T*_) [75]. Briefly, *C*_*T*_ data for each sample was normalized against its corresponding 16S RNA *C*_*T*_ prior to log transformation (2^*-ΔC*^_*T*_). At least 5 replicates were included in the analysis.

### Preparation of Bacterial Extracts for Quantitative Proteome Studies

*P. gingivalis* strains W83 and W83Δ717 were grown on sBAP for two passages of 48 h each before culturing in sTSB. Bacteria from sBAP cultures were transferred to sTSB and grown to early stationary phase (approximately 18 h). Bacterial concentrations in all cultures were determined by optical density readings performed at 550 nm. For proteome studies, bacterial numbers of each inoculate were adjusted in fresh sTSB to match the same concentration. Bacterial numbers of each inoculate were confirmed by culture.

For protein extraction, bacterial cultures from 3 independent experiments were pelleted by centrifugation at 15000 × *g* for 10 min at 4°C. Pelleted cultures were washed three times with sterile phosphate buffered saline and stored frozen at -80°C before processing. Protein from frozen pellets was extracted with BugBuster™ Master Mix protein extraction reagent (Novagen®, EMD4Biosciences, Gibbstown, NJ, USA) supplemented with Protease Inhibitor Cocktail Set III (Calbiochem, EMD4Biosciences) according to the manufacturer’s instructions. Total protein concentrations from extracts were determined with Non-Interfering Protein Assay Kit (Calbiochem).

### Quantitative Proteomic Analysis using Peptide-Labeling and Offline 2D-LC-MS/MS

W83 and W83Δ717 *P. gingivalis* protein extracts from 3 independent experiments were pooled for analysis. Pooled extracts were processed and labeled with an amine-specific peptide-based labeling system iTRAQ™ according to the manufacturer’s instructions (AB Sciex, Foster City, CA, USA). Briefly, a 60-µg protein pellet was dissolved in 20 µL of dissolution buffer (0.5 M triethylammonium bicarbonate) and reduced with reducing agent (50 mM tris-2-carboxyethyl phosphine) at 60°C for 1 h. After reduction, sulfhydryl groups on cysteine residues were blocked with 200 mM methyl methanethiosulfonate for 10 min at room temperature. Ten microliters of a trypsin solution (Promega Corporation, Madison, WI, USA) was added to each sample and incubated overnight at 37 °C. After digestion, protein extracts from each sample were labeled and combined. Pooled samples were desalted by using a macrospin column Vyadac Silica C_18_ (The Nest Group Inc, Southboro, MA, USA) prior to strong cation exchange (SCX) procedure.

SCX fractionation of desalted iTRAQ labeled peptides was performed with a polysulfoethyl A column (100 × 2.1 mm, 5 µm, 300 Å). Peptides suspended in buffer A (75% 0.01 M ammonium formate, 25% acetonitrile) were eluted during a linear gradient of 0-20% buffer B (75% 0.5 M ammonium formate, 25% acetonitrile) and detected at an absorbance of 280 nm. Eluted fractions were further separated by capillary reverse phase HPLC using an LC Packing C_18_ Pep Map column (DIONEX, Sunnyvale, CA). Mass spectrometric analysis of column elute was performed inline with a hybrid quadrupole-TOF mass spectrometer QSTAR (Applied Biosystems). The focusing potential and ion spray voltage was set to 275 V and 2600 V, respectively. The information-dependent acquisition mode of operation was employed in which a survey scan from *m/z* 400–1200 was acquired followed by collision induced dissociation of the three most intense ions.

### Protein Identification from Mass Spectra

Tandem mass spectra were extracted by Analyst (v 1.1; Applied Biosystems/ MDS Sciex). Concatenation of the forward and random sequences from the W83 protein sequence (National Center for Biotechnology Information, Bethesda, MD) was used for protein identification. Protein identification searches were performed using MS/MS data interpretation algorithms from Protein Pilot™ (Paragon™ algorithm, v 2.0, AB Sciex) [76]. The confidence level for protein identification was set at 80% for peptide score, with a minimum of 2 peptides per protein identification and 95% for protein probability. False discovery rate was calculated using a decoy search. Protein ratios were generated with Pro Group™ algorithm and automatically corrected for bias. Protein quantification was performed for proteins detected with a minimum of two spectra within the experiment. Only protein ratios with an error factor (EF) < 2 were retained for further analysis. EF is a measure of the variation among the different iTRAQ™ ratios (the greater the variation, the greater the uncertainty) and represents the 95% uncertainty range for a reported ratio. The calculated *P*-value obtained with the ProGroup™ algorithm is based on 95% confidence interval and protein ratios with *P* <0.05 were considered significant. Gene ontology information for each identified protein was obtained from Uniprot (www.ebi.ac.uk/uniprot) [77] or TIGR (cmr.jcvi.org/tigr-scripts).

### Statistical analysis

Experiments with 3 or more biological replicates were analyzed with Statview Version 5.0.1 software (SAS Institute, Cary, NC). ANOVA followed by Fisher’s multiple comparison test. For all analyses a probability of P < 0.05 was considered significant.

## Supporting Information

File S1 **Temporal gene expression profile of PG0717 during invasion of HCAEC; Construction, Validation, and Phenotypic Characterization of the W83Δ717 Mutant.**
(PDF)

File S2 **Vector controls, co-localization of Rab5 with *P. gingivalis.***
(PDF)

File S3 **Comparative Proteome Data of W83Δ717 versus W83.**
(XLS)
