# Supplementary material for: Deletion of Lipoprotein PG0717 in Porphyromonas gingivalis W83 Reduces Gingipain Activity and Alters Trafficking in and Response by Host Cells
Source: PLoS One. 2013 Sep 12;8(9):e74230. doi: 10.1371/journal.pone.0074230 (PMC3772042; doi:10.1371/journal.pone.0074230)
Supplement: File S1 — Temporal gene expression profile of PG0717 during invasion of HCAEC; Construction, Validation, and Phenotypic Characterization of the W83Δ717 Mutant. (PDF) [file pone.0074230.s001.pdf]

Supporting File 1.

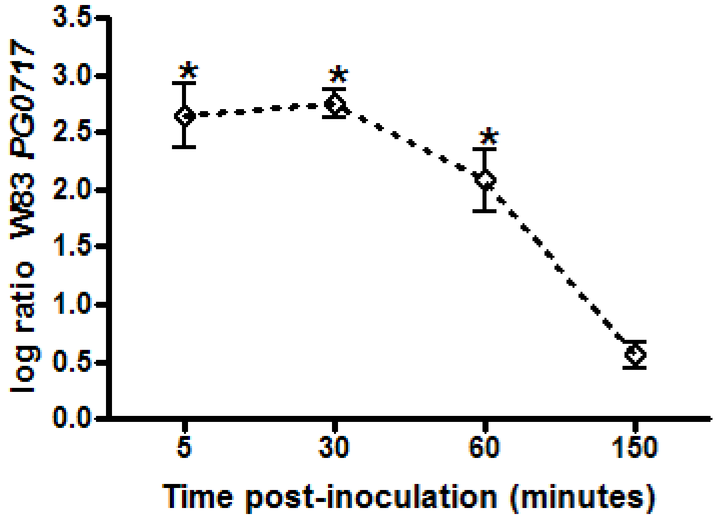

Figure S1A. The temporal expression of *P. gingivalis PG0717*during invasion of HCAEC. Invasion experiments were performed as previously described [Rodrigues and Progulske-Fox, 2005]. Adhered or internalized W83 was harvested from HCAEC cultures at 5 min, 30 min, 1 h, and 2.5 h post- inoculation and processed for microarray analysis as already described. Briefly, total RNA from W83 broth cultures (prior to invasion) and internalized bacteria was extracted by using 10 ml of Trizol reagent followed by RNA isolation as described by the manufacturer (Invitrogen Life Technologies, Carlsbad, CA). All RNA samples were DNase treated and purified using the RNeasy kit (QIAGEN Inc., Valencia, CA). To separate bacterial total mRNA from poly(A) mRNA, cellular and internalized bacterial RNAs were also treated with the Oligotex kit (QIAGEN) according to the manufacturer's instructions and the supernatant (invasion RNA) was again treated with Trizol LS reagent (Invitrogen Life Technologies). Reverse transcription (RT) and microarray reactions were performed either with 2.0 µg of total bacterial RNA (control) or with invasion RNA (200 µg of total RNA containing 2.0 µg of bacterial RNA), collected from one T-75 flask of invaded HCAEC (per microarray slide), as previously described Details of the microarrays can be found at http://www.tigr.org. The resulting images were analyzed by TIGR Spotfinder 1.0 and TIGR Multiple Experiment Viewer software 1.2 (The Institute for Genomic Research [TIGR] [http://www.tigr.org]). The generated files were imported into Microsoft Excel (Microsoft Corporation, Redmond, WA) for subsequent analyses. Gene expression values were log transformed prior to statistical analysis by ANOVA. The results represent the mean log ratio ± SD of three independent biological replicate arrays performed with three different RNA samples. * Mean ratios were statistically different from control ($P < 0.005$).

Construction and validation of W83Δ717

Figure S1B. Gene context of *PG0717* with orientation and approximate position of *erm* insertion

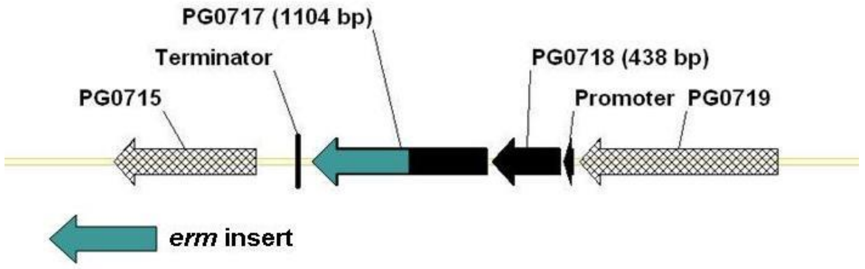

Table S1: Primers used for mutant construction, sequencing, and Northern blot analysis

**Construction**	**A fragment - forward**	GGGAGCGCGATGGTAAC
	**A fragment - reverse**	CAGTGCGAGTCTAGATGACAA
	**B fragment - forward**	TTCCATGGCTCCCTTTTCTACTTC
	**B fragment - reverse**	GCCCCGATATTGCGTCAC
**Sequencing**	**Erm - forward**	5'-AAACACGCCAAAGTAAACAATTTA
	**Erm- reverse**	5'-CCGTATCCTGATTACTTATATTTGC
**Northern probes**	**717 - forward**	5'-AAAGGGAGACCAGGAAGTCGACTTGTTCTA
	**717 - reverse**	5'-TTGTTTTCGTATGCATCATCATCGTAGTCA
	**718 - forward**	5'-TGCTAAGGTATCATACGTAAAGATGGACGA
	**718 - reverse**	5'-CACTTCATAGTCTCTACTATCCCGATCGAT

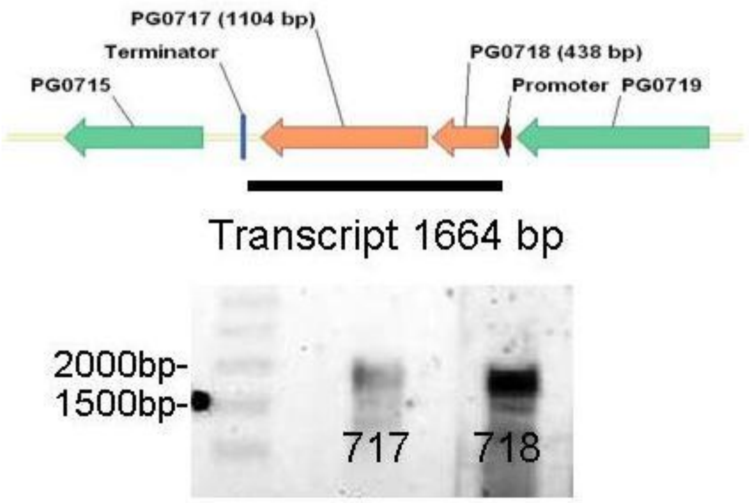

Figure S1C. Northern blot analysis of *PG0717* and *PG0718* in *P. gingivalis* W83 *P. gingivalis* was grown to early stationary phase and total RNA was extracted as described in methods. Ten micrograms of total RNA from W83 was loaded into each lane. PG0717 and PG0718 mRNA was identified with biotin labeled probes that were constructed using primers listed in Table S1.

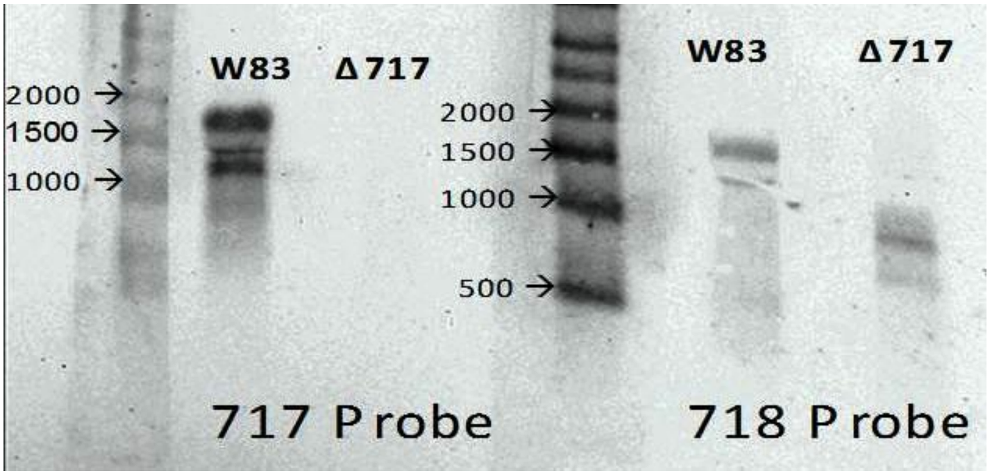

Figure S1D. Northern Blot analysis of W83 and W83Δ717 (Δ717) *P. gingivalis* strains were grown to early stationary phase and total RNA was extracted as described in methods. Ten micrograms of total RNA from W83 and W83Δ717 was loaded into each lane. PG0717 and PG0718 mRNA was identified with biotin labeled probes that were constructed using primers listed in Table S1.

Figure S1E. Sequencing of the genome region flanking *PG0717* in W83Δ717

Results of blast analysis of ERM sequence data:

```
PG717 forward erm sequence
Features in this part of subject sequence:
   putative lipoprotein

 Score =  407 bits (220),  Expect = 4e-114
 Identities = 235/242 (97%), Gaps = 2/242 (0%)
 Strand=Plus/Minus

Query  162     GATGACAAAAAATAAAGTTATATGATCTGACAACACTTTTTTCAGGAGGAGCCGACAGCC  221
               ||||||||||||||||||||||||||||||||||||||||||||||||||||||||||||
Sbjct  770095  GATGACAAAAAATAAAGTTATATGATCTGACAACACTTTTTTCAGGAGGAGCCGACAGCC  770036

Query  222     CTCTGAATGAAGTTAGCATAAAGAACAGAGGGCGATCCAAATATTCTTGGGTCGCCCTCT  281
               ||||||||||||||||||||||||||||||||||||||||||||||||||||||||||||
Sbjct  770035  CTCTGAATGAAGTTAGCATAAAGAACAGAGGGCGATCCAAATATTCTTGGGTCGCCCTCT  769976

Query  282     TCTTTTTTGCAGGATGAGTACATTTGCCGAT-GCTTCAGTGCAAAAACAATGGTTTCTGA  340
               ||||||||||||||||||||||||||||||| ||||||| |||||||||||||| |||||
Sbjct  769975  TCTTTTTTGCAGGATGAGTACATTTGCCGATAGCTTCAGAGCAAAAACAATGGTCTCTGA  769916

Query  341     ATGCAAAAAATCTTTTTCGTTTGCGTGA-AATCTTACCTTTGTGGAGCTGATTTTTTCAG  399
               |||||||  ||||||||||||||||||| ||||||||||||||||||||||||||||| |
Sbjct  769915  ATGCAAAGCATCTTTTTCGTTTGCGTGATAATCTTACCTTTGTGGAGCTGATTTTTTCGG  769856

Query  400     CA  401
               ||
Sbjct  769855  CA  769854
```

PG717 Reverse erm sequence

```
Porphyromonas gingivalis W83, complete genome
Length=2343476

 Features in this part of subject sequence:
   putative lipoprotein

 Score =  263 bits (142),  Expect = 6e-70
 Identities = 145/146 (99%), Gaps = 1/146 (0%)
 Strand=Plus/Plus

Query  390     TGG-CTCCCTTTTCTACTTCTATGACATAGAATATTTCGGTACCGTTACGTTCTACCTTA  448
               ||| ||||||||||||||||||||||||||||||||||||||||||||||||||||||||
Sbjct  770793  TGGTCTCCCTTTTCTACTTCTATGACATAGAATATTTCGGTACCGTTACGTTCTACCTTA  770852

Query  449     TCTATGTCTTCTATTTTCCACTTGGCATATTCACTTGCCTCAAAAGCAGCTCGGACTGCT  508
               ||||||||||||||||||||||||||||||||||||||||||||||||||||||||||||
Sbjct  770853  TCTATGTCTTCTATTTTCCACTTGGCATATTCACTTGCCTCAAAAGCAGCTCGGACTGCT  770912

Query  509     TTAGGCAGGGCGCTGTAGGGAATGTC  534
               ||||||||||||||||||||||||||
Sbjct  770913  TTAGGCAGGGCGCTGTAGGGAATGTC  770938
```

Figure S1F. Comparison of W83 and W83Δ717 growth rates in sTSB broth.

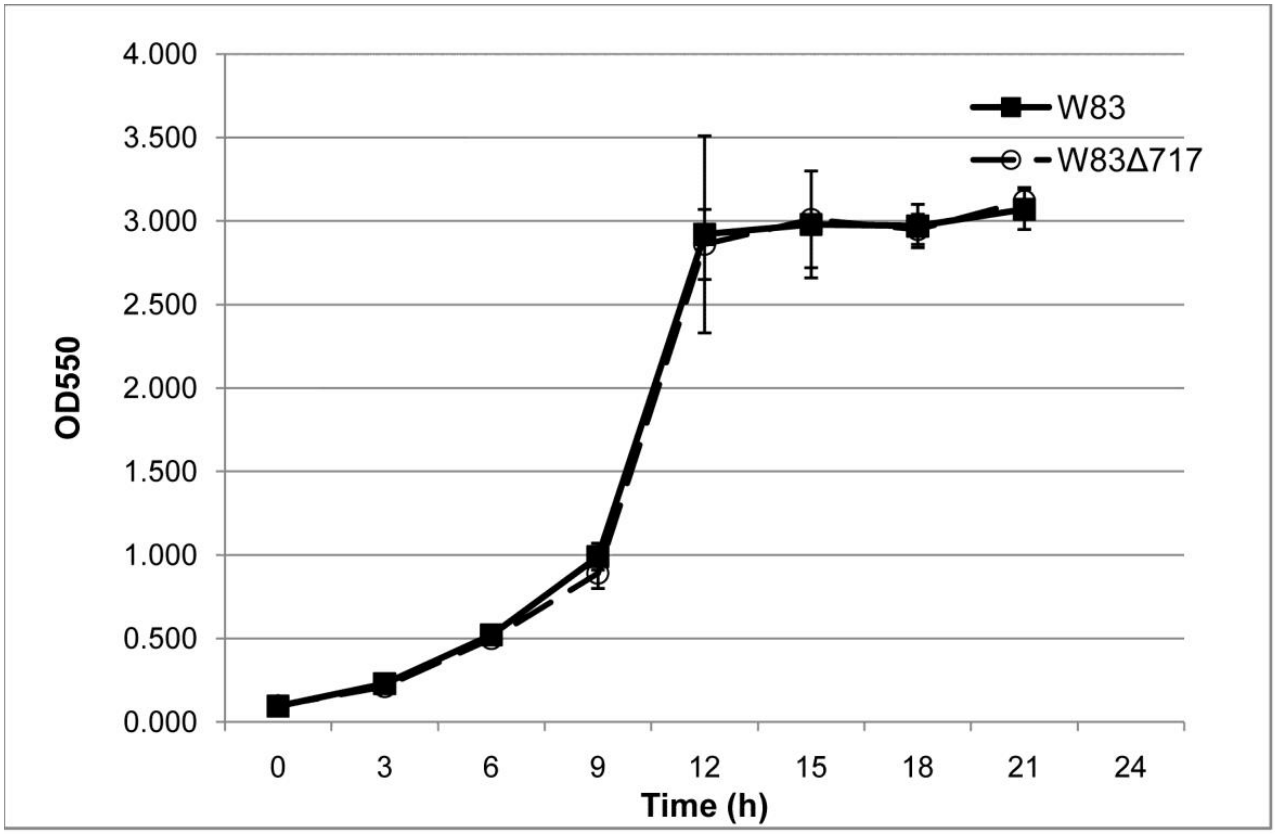

Comparison of the growth of W83 and W83Δ717 was carried out in liquid cultures inoculated with an overnight (18-h) liquid culture of each organism at on OD550 of 0.10 ± 0.01. Growth was monitored spectrophotometrically at OD550 on a SmartSpec Plus spectrophotometer (Bio-Rad, Hercules, CA, USA) every 3 h for 21 h. Values represent the mean ± SD of 3 biological replicates.

W83 sample B

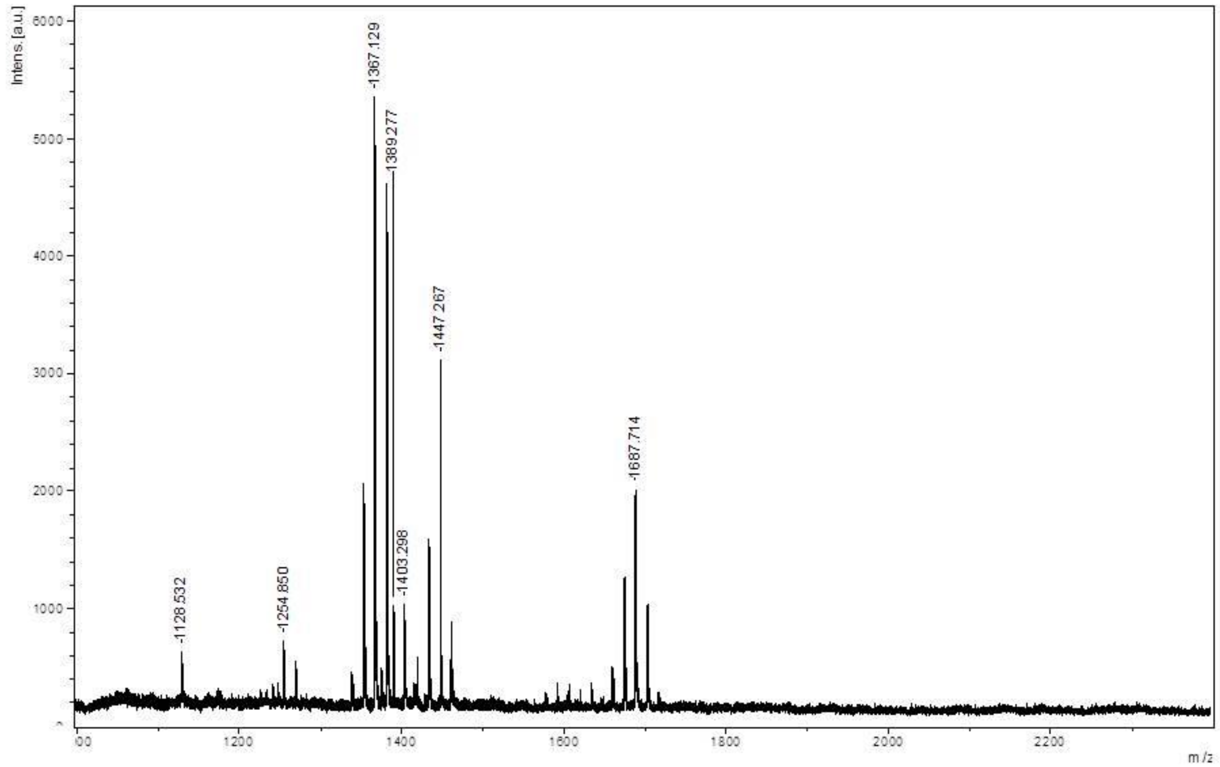

W83Δ 717 sample B

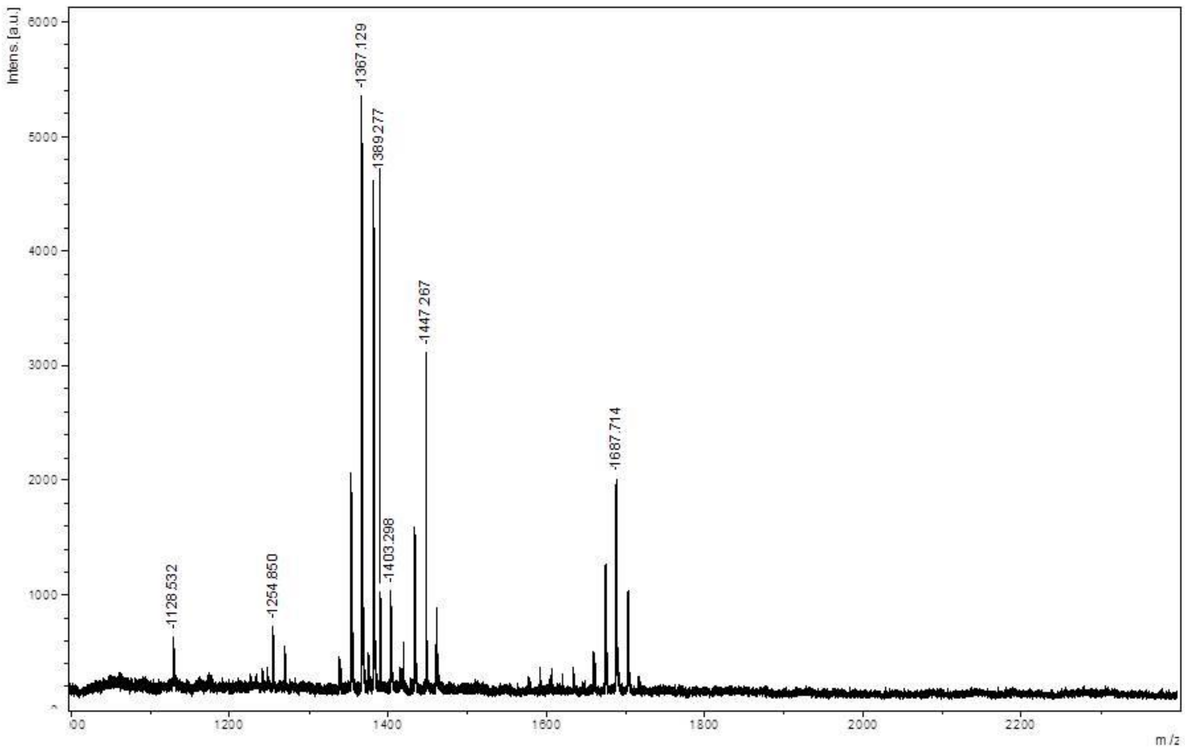

Figure S1G. The composition of the lipid A molecule is not significantly altered upon deletion of PG0717. MALDI-TOF/MS of purified lipid A from W83 (panel A) and the 717 deletion mutant (panel B).

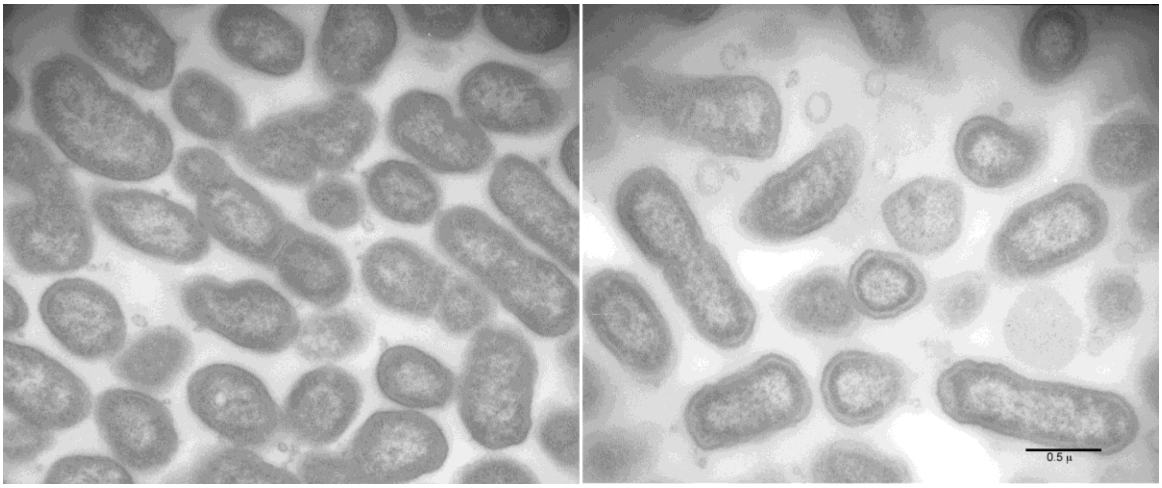

Figure S1H. Electron micrographs of W83 and W83Δ717 after staining with ruthenium red. Images are representative of 2 independent sets of stained culture preparations, minimum 3 images per strain per set. Bar, 500 nm.

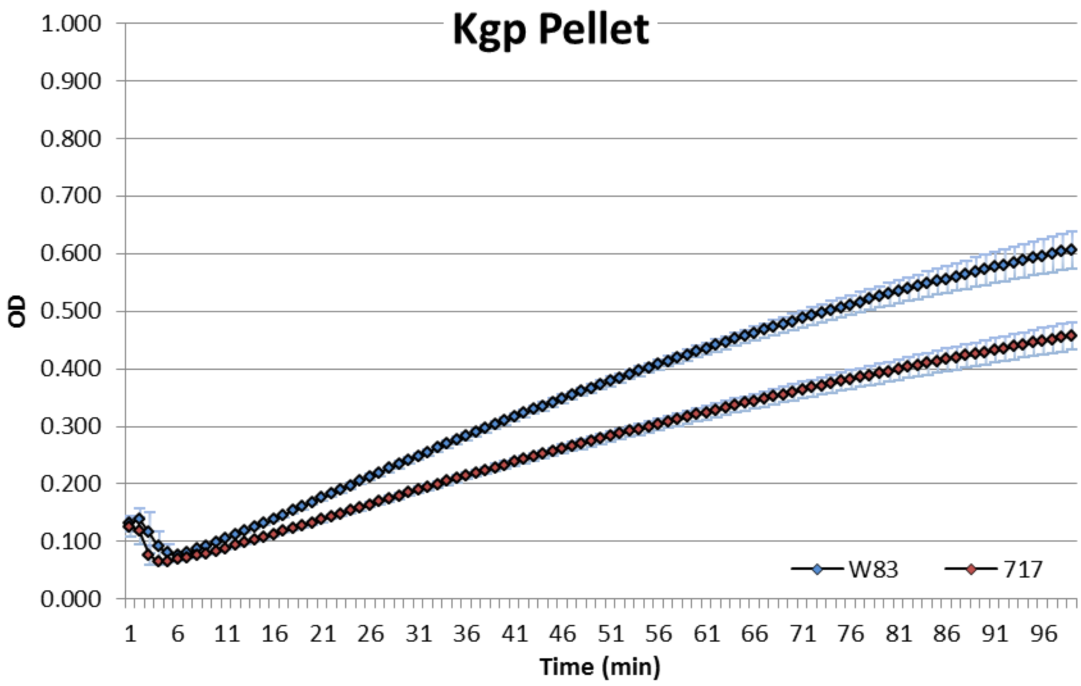

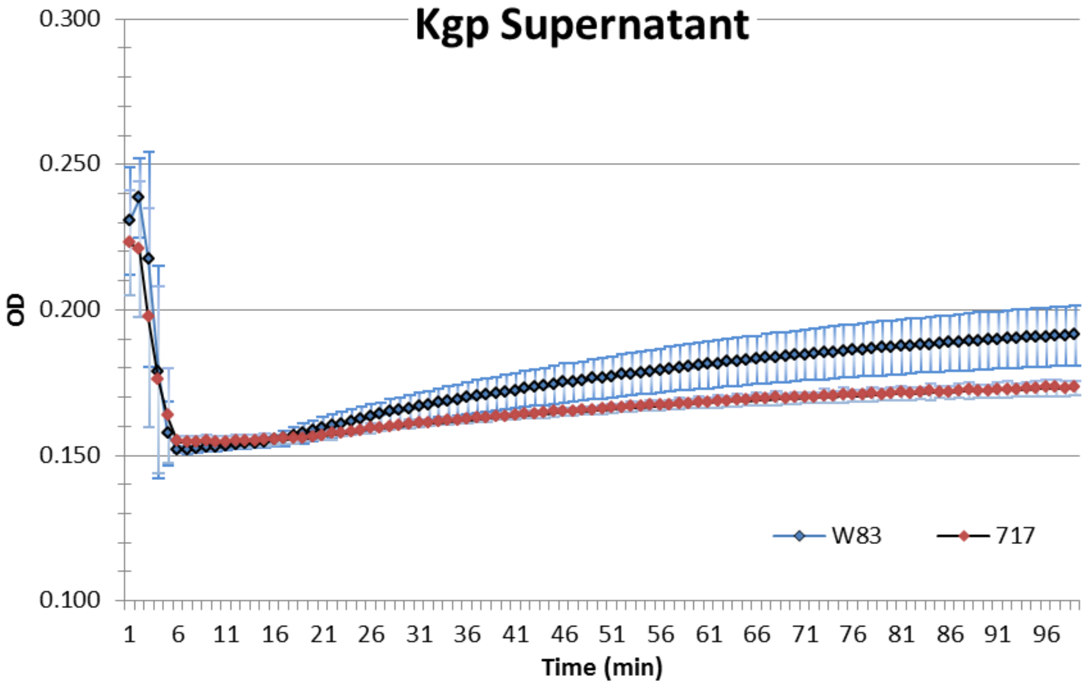

Figure S1I. Lysine (KGP, upper panels) and arginine (RGP, following page) gingipain activity over time. Experiments were carried out on bacterial cells ("Pellet") and culture supernatants ("Supernatant") of both W83 and W83Δ717 as described in Materials and Methods. Graphs shown are the average ±SD of two independent determinations. Slopes from the linear portions of the curves were used to calculate the activity levels reported in Table 1.

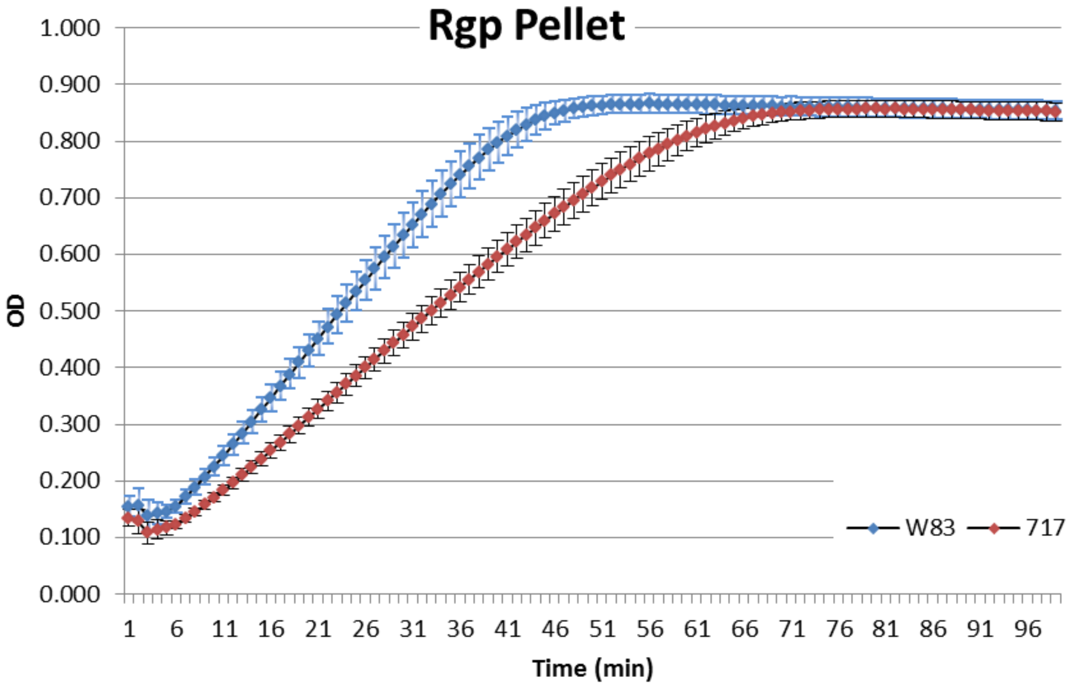
Rgp Pellet
OD
1.000
0.900
0.800
0.700
0.600
0.500
0.400
0.300
0.200
0.100
0.000
1 6 11 16 21 26 31 36 41 46 51 56 61 66 71 76 81 86 91 96
Time (min)
W83
717

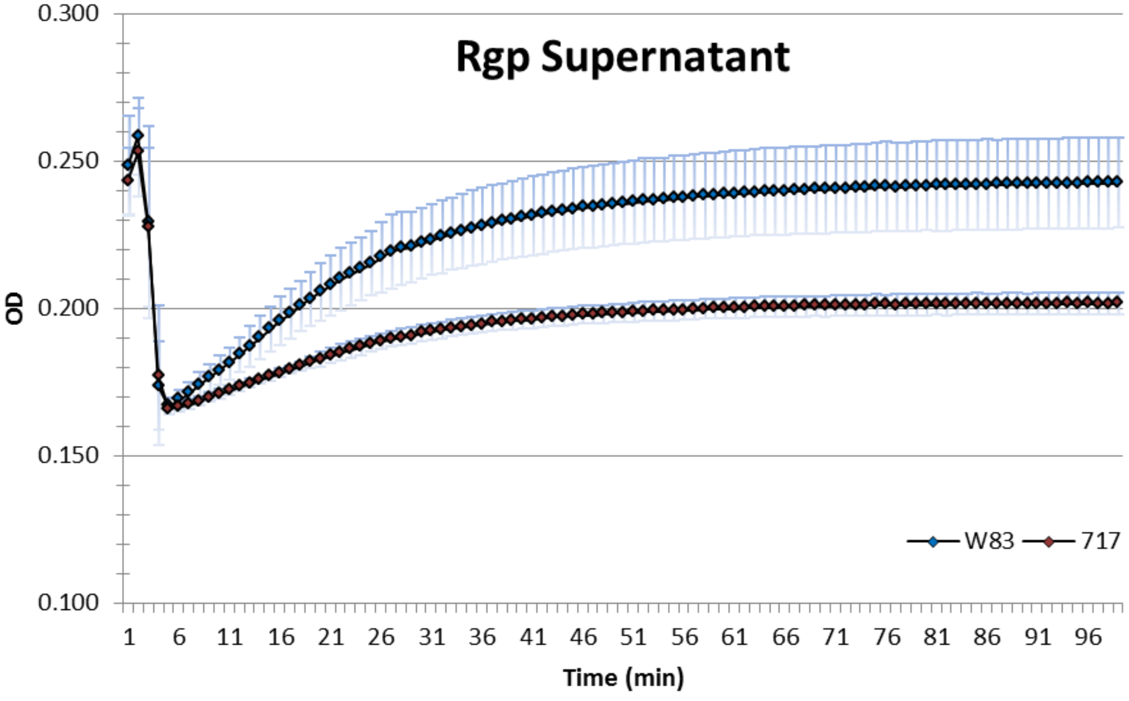
Rgp Supernatant
OD
0.300
0.250
0.200
0.150
0.100
1 6 11 16 21 26 31 36 41 46 51 56 61 66 71 76 81 86 91 96
Time (min)
W83
717
