# Supplementary material for: Deletion of Lipoprotein PG0717 in Porphyromonas gingivalis W83 Reduces Gingipain Activity and Alters Trafficking in and Response by Host Cells
Source: PLoS One. 2013 Sep 12;8(9):e74230. doi: 10.1371/journal.pone.0074230 (PMC3772042; doi:10.1371/journal.pone.0074230)
Supplement: File S2 — Vector controls, co-localization of Rab5 with P. gingivalis. (PDF) [file pone.0074230.s002.pdf]

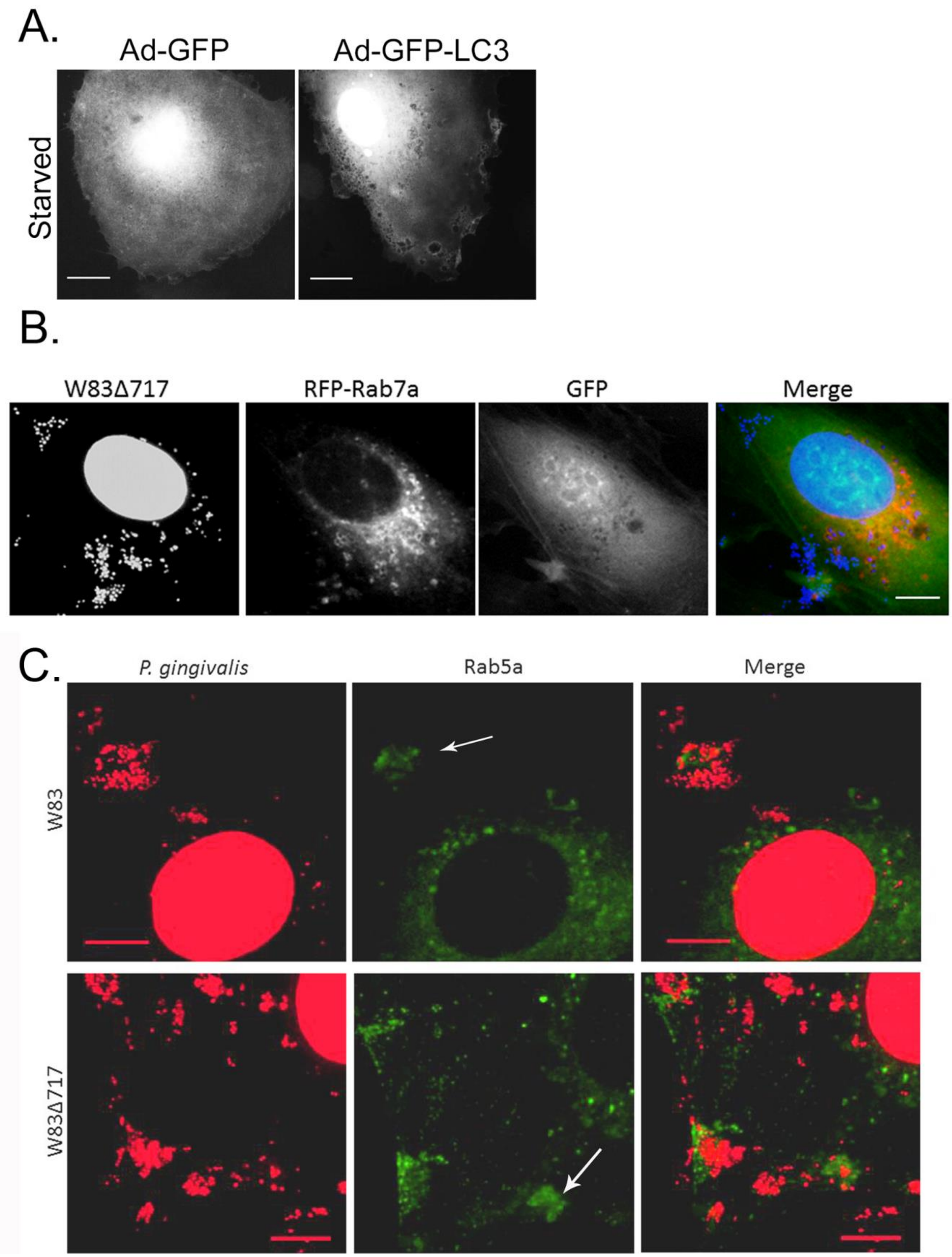

Figure S2. Representative images of HCAEC transduced with Adenovirus (Welgen, Inc. Worcester, MA) or Bacmam 2.0 (Life Technologies™) vectors. (A) Starved HCAEC transduced with Ad-GFP or Ad-GFP-LC3. (B) Fed W83Δ717 infected HCAEC transduced with Bacmam GFP and RFP-Rab7a vector. (C) Fed W83 and W83Δ717 (red) infected HCAEC that were transduced with RFP-Rab5a (green). Scale bar is equivalent to 10 μm.
